# Reverse Axillary Mapping in Breast Cancer Using Blue Dye: A Tertiary Setup Experience

**DOI:** 10.7759/cureus.18576

**Published:** 2021-10-07

**Authors:** Arun H Narasannaiah, Ali Z Anwar, Manjunath KV, Yeshwanth R, Syed Althaf, Praveen Arakeri, Siddharth Jain, Rajalakshmi S Kumar, Mohammed A Ali, Nikhil Manukonda

**Affiliations:** 1 Surgical Oncology, Kidwai Memorial Institute of Oncology, Bangalore, IND

**Keywords:** reverse axillary mapping, india, blue dye, general surgery and breast cancer, breast cancer research

## Abstract

The concept of reverse axillary mapping originated with the main purpose of reducing lymphedema. In this study, we test the advantage of reverse axillary mapping to delineate the arm-draining lymph nodes and their involvement in various stages of breast carcinoma. In this study, we also attempt to redefine the template for axillary dissection in breast cancer.

During the period of September 30, 2020, to August 30, 2021, 46 patients were recruited to undergo a procedure in which isosulfan blue dye was injected into the upper arm and the axilla was explored to isolate the lymph nodes. The lymph nodes were submitted for examination histopathologically.

The results conclusively showed that axillary lymph node metastasis was only influenced by the advanced stage of the disease (p=0.014) and the visualization of the lymphatics was independent of the stage, type of surgery, decubitus, or age.

The study conclusively shows that attempts to preserve the upper limb-draining nodes in advanced stages would be futile and the preservation of such lymph nodes should be limited to the early stages of breast cancer.

## Introduction

In the management of operable breast cancer, the axillary lymph node status is the most important prognostic factor. The standard approach for axillary lymph node staging apart from imaging is surgical by either sentinel lymph node biopsy (SLNB) or axillary lymph node dissection (ALND). Among the complications associated with SLNB and ALND, upper limb lymphedema is the most distressing, occurring in 7% and 30% [1-2]. In reverse axillary mapping, the hypothesis is that the upper limb lymphatics are delineated by blue dye injected into the medial arm during SLNB or ALND, which can be preserved, thereby preventing upper limb lymphedema.

The lymph node draining the upper limb appears as a blue node called the axillary reverse mapping (ARM) node and has been reported in the early studies as not harboring metastatic disease [3-5]. Some studies like Ponzone et al. reported that approximately 10% of patients with extensive nodal diseases had ARM node involvement [6].

Hence, this preliminary analysis of ARM using blue dye is done to study the visualization, define the anatomy of upper limb lymphatics in the axilla, and identify and define the anatomical location of the ARM node in the axilla and its involvement by metastasis in breast cancer.

## Materials and methods

The study included women diagnosed with breast cancer from September 30, 2020, to August 30, 2021.

Inclusion criteria were as follows: patients planned for breast conservation surgery (BCS) or modified radical mastectomy (MRM); patients aged over 18 years; patients willing to participate in the study; patients with operable breast cancer (T1-4b, N0-1, M0) upfront or post-neoadjuvant (chemo or hormone) therapy; and patients undergoing ALND post lumpectomy.

Exclusion criteria were as follows: patients aged less than 18 years; patients not willing to participate; patients who were allergic to the dye; patients with T4c, T4d, N2-3, and M1 disease undergoing palliative mastectomy.

A total of 46 patients were included in the study; their data were collected and preliminarily analyzed.

Procedure

With prior informed consent, 3-5 ml isosulfan blue dye was injected intradermally at the sulcus between the triceps and biceps of the arm before induction of anesthesia after the test dose. A gentle massage was done at the injection site and arm exercises were done for a few minutes. On entering the axillary opening, clavipectoral fascia, careful dissection was done at the superior lateral aspect 2-3 cm below the axillary vein to identify the blue lymphatics and node. The anatomical location of blue lymphatics and node is documented and the lymph nodes were sent for histological examination separately, after completion of ALND.

Pathological examination

All of the nodes harvested in ALND were sampled after being cut into 2 mm slices in the longitudinal axis and submitted to microscopic examination with hematoxylin and eosin. The ARM nodes (number and metastases) were studied and reported separately.

Statistical analysis

Data were categorized into various variables like age, TNM (Tumor Node Metastasis) stage, type of surgery performed, the location of ARM nodes, and the histological nature of ARM nodes. The correlation analysis was done using the chi-square test between the variables of age, TNM stage, histology of node, and breast surgery. Clearance was taken from the medical ethics committee (Institutional Review Board) of the institution (Kidwai Memorial Institute of Oncology) with reference identification KMIO/MEC/017/31.August.2020.

## Results

**Figure 1:**
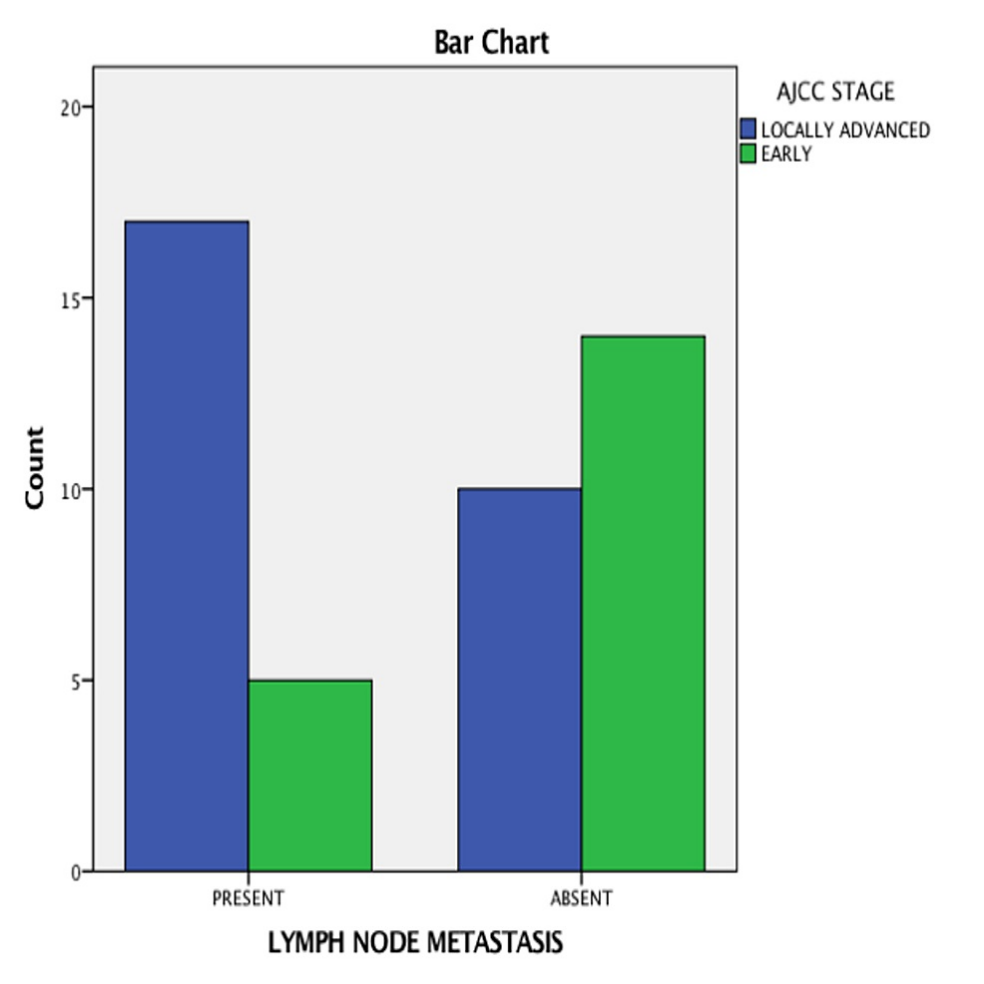
Lymph node metastasis seen in the axillary reverse mapping (ARM) node as compared to the American Joint Committee on Cancer (AJCC) stage

**Figure 2:**
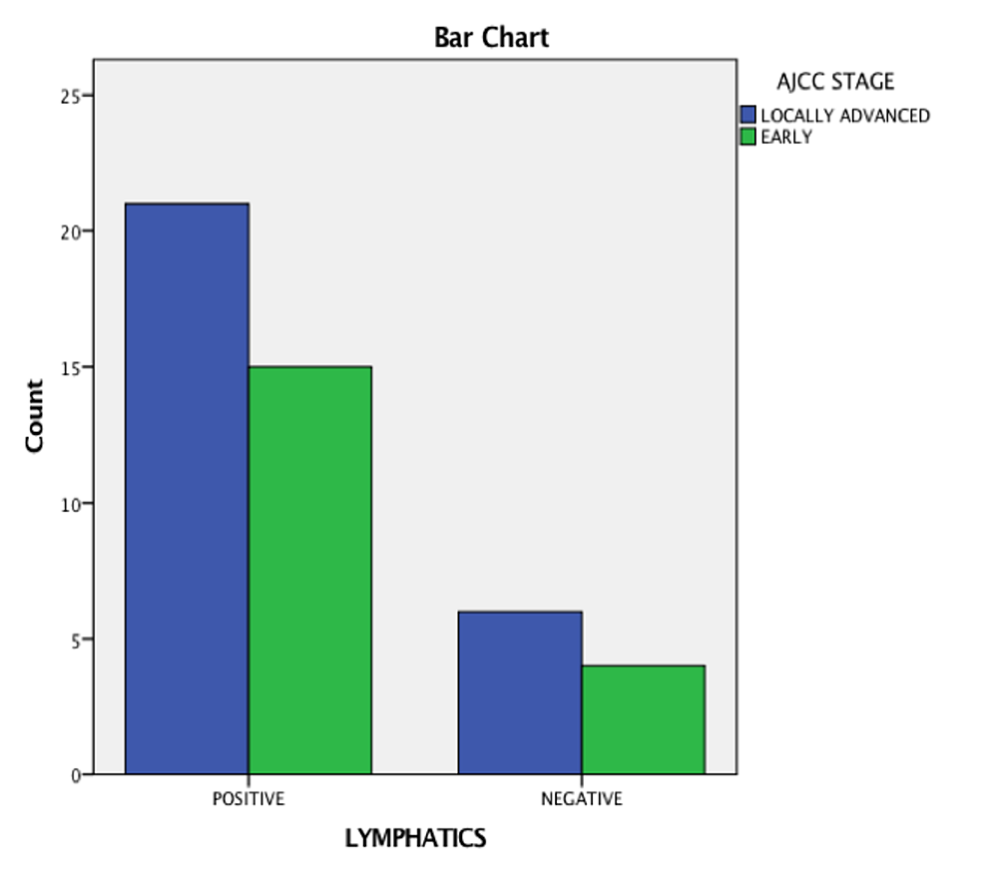
Visualization of lymphatics in relation to the American Joint Committee on Cancer (AJCC) stage

There were 46 cases selected for analysis between the period from September 2020 to August 2021. They were grouped based on tumor stage, age, visualization of lymph node, ARM node, identification, and pathological involvement. The total number of patients who underwent the study was 46 with a median age of 45.2 years. Their baseline characteristics are described in Tables 1-3. The baseline characteristics of patients with respect to the involvement of the ARM node are shown in Table 4 and with respect to the American Joint Committee on Cancer (AJCC) stage are shown in Figure 1. The baseline characteristics of patients with respect to the visualization of lymphatics are shown in Table 5 and with respect to the AJCC stage are shown in Figure 2.

**Table 1:** Tumor stage (T) according to the American Joint Committee on Cancer (AJCC) T1- tumor size ≤20 mm; T2- tumor size 20 mm to ≤50 mm; T3- tumor size >50 mm; T4- tumor with infiltration into the chest wall, skin, ulceration or both, inflammatory carcinoma

Tumor stage	Number (percentage of total)
T1	3(6.5%)
T2	22(47.8%)
T3	17(36.9%)
T4	4(8.6%)
Total	46

**Table 2:** Nodal stage (N) according to the American Joint Committee on Cancer (AJCC) N1 - metastasis to ipsilateral level 1, 2 axillary node; N2 - metastasis to matted ipsilateral level 1, 2 axillary node or metastasis to ipsilateral internal mammary nodes; N3 - metastasis to ipsilateral level 3 axillary node or metastasis to ipsilateral supraclavicular node or metastasis to both ipsilateral level 1, 2 axillary node and ipsilateral internal mammary nodes

Nodal stage	Number (percentage of total)
N0	7(15.2%)
N1	23(50%)
N2	15(32.6%)
N3	1(2.1%)
Total	46

**Table 3:** American Joint Committee on Cancer (AJCC) stage of the various patients involved

AJCC stage	Number(percentage of total)
1	1(2.1%)
2A	6(13.04%)
2B	12(26.08%)
3A	22(47.8%)
3B	4(8.6%)
3C	1(2.1%)
Total	46

**Table 4:** Characteristics of the patient as compared to the involvement of the axillary reverse mapping (ARM) nodes AJCC: American Joint Committee on Cancer; BCS: breast conservation surgery; MRM: modified radical mastectomy

	Number of patients	ARM node-positive	ARM node-negative	Chi-square	P-value	Odds ratio (95%CI)
AJCC stage				6.002	0.014	4.76(17.21-1.31)
Early	19	14	5			
Locally advanced	27	10	17			
Surgery				0.987	0.331	1.813(6.04-0.54)
BCS	18	7	11			
MRM	28	15	13			
Decubitus				2.14	0.14	0.41(1.35-0.12)
Right	22	13	9			
Left	24	9	15			
Age				0.19	0.65	0.75(2.65-0.21)
≥50	14	6	8			
<50	32	16	16			

**Table 5:** Visualization of the lymphatics as compared to the patient characteristics AJCC: American Joint Committee on Cancer; BCS: breast conservation surgery; MRM: modified radical mastectomy

	Number of patients	Lymphatics seen	Lymphatics not seen	Chi-square	P-value	Odds ratio (95%CI)
AJCC stage				6.002	0.014	4.76(17.21-1.31)
Early	19	15	4			
Locally advanced	27	10	17			
Surgery				0.447	0.50	0.60(2.70-0.13)
BCS	18	15	3			
MRM	28	21	7			
Decubitus				0.75	0.38	1.87(7.80-0.45)
Right	22	16	6			
Left	24	20	4			
Age				0.552	0.45	0.57(2.48-0.13)
≥50	14	10	4			
<50	32	26	6			

## Discussion

In the management of breast carcinoma, the axillary nodal status has been shown to have a prognostic effect in both the early and locally advanced status of the disease. Although the management of axillary nodes has drastically changed over time, including progression to a more conservative approach - from complete axillary nodal removal to the preservation of uninvolved nodes.

Axillary reverse mapping was propounded in lieu of such an approach, which includes the identification and preservation of limb-draining lymphatics with the help of a dye. Hence, in this study, we attempt to identify limb-draining lymphatics using isosulfan dye and ascertaining their involvement with the disease. Table 6 demonstrates the salient features of various studies that attempted reverse axillary mapping.

**Table 6:** Comparison of the ARM procedure between various studies ARM: axillary reverse mapping

Study	Number of ARM procedures	Rate of identification	Ratio of metastatic involvement	Technique
Thompson et al. [7]	18	61%	0/7	Blue dye
Casabona et al. [3]	9	88.9%	0/3	Blue dye
Nos et al. [4]	21	71%	0/10	Blue dye
Boneti et al. [5]	47	40.6%	0/15	Blue dye
Ponzone et al. [6]	49	73.5%	3/27	Blue dye
Bedrosian et al. [8]	30	70%	2/	Blue dye
Deng et al. [9]	69	NR	6/69	Blue dye
Han et al. [10]	97	Nr	2.17	Blue dye
Kang et al. [11]	129	78.3%	9/101	Blue dye
Rubio et al. [12]	36	83.3 %	4/30	Blue dye
Gobardhan et al. [13]	93	90.3%	11/93	Blue dye
Connor et al. [14]	60	75%	3/19	Blue dye
Schunemann et al. [15]	45	88.9%	10/45	Blue dye
Ochoa et al. [16]	127	75%	5/27	Blue dye
Our study	46	73.2%	24/46	Blue dye

As seen in this study, the rate of identification using blue dye was 73.2% %; up 40% to 90% when compared to other studies. In the case series of Gobardhan et al. [13] and Schunemann et al. [15], a high detection rate was encountered of up to 90% where a similar technique was used. Further comparison is summarized in Table 6.

**Figure 3:**
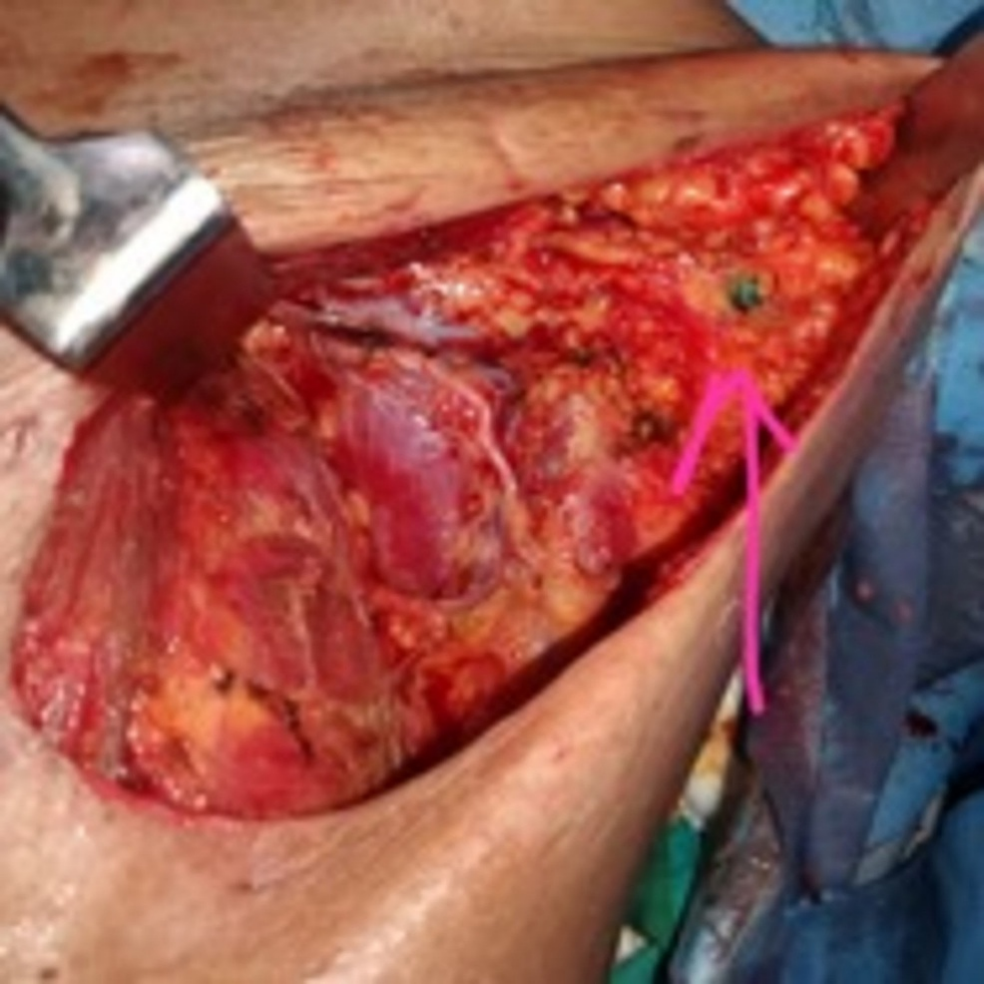
Visualized ARM node stained with blue dye (pink arrow) ARM: axillary reverse mapping

The visualization of the lymphatics remained independent of the status of the disease, showing no significant association between them, ascertaining that the visualization of the lymphatics was unimpeded in all cases, with a high success rate. The location of the lymph nodes draining the upper limb was generally found predominantly inferior to the axillary vein situated laterally to the thoracodorsal pedicle, which was similarly seen in Kumar et al. [17]; however, in a minority of cases, the draining lymph nodes were also found superior to the axillary vein and medial to the thoracodorsal pedicle. Figure 3 shows the visualized ARM node.

There was no correlation found between the decubitus of the diseases or type of surgery used, breast conservation, or radical to the positivity of the limb draining lymph nodes. There was also no significant relation with the visualization of the lymphatics.

In a separate study [15], a correlation was found between the positivity rate of metastasis found in upper limb draining nodes and age; however, no such relation was found in this study, indicating that age had no effect on the positivity of the node

There was a significant association between a high tumor burden and ARM node involvement, which includes high tumor status, high nodal status, and advanced stage of the disease as similarly seen in Schnuemann et al. [15]. In other studies, the participants had a mild burden of disease, showing only minimal nodal involvement [2,4], hence the limb draining lymph nodes had minimal involvement.

In studies that included patients with more varied data [6,12,14,16], a similar association of limb draining nodes harboring the disease was seen.

The visualization of the lymphatics remained independent of the burden of the disease, as no significant association was found between them. Hence, it can be postulated that the lymphatics could be traced using the given dye even in the varied nature of cases.

**Figure 4:**
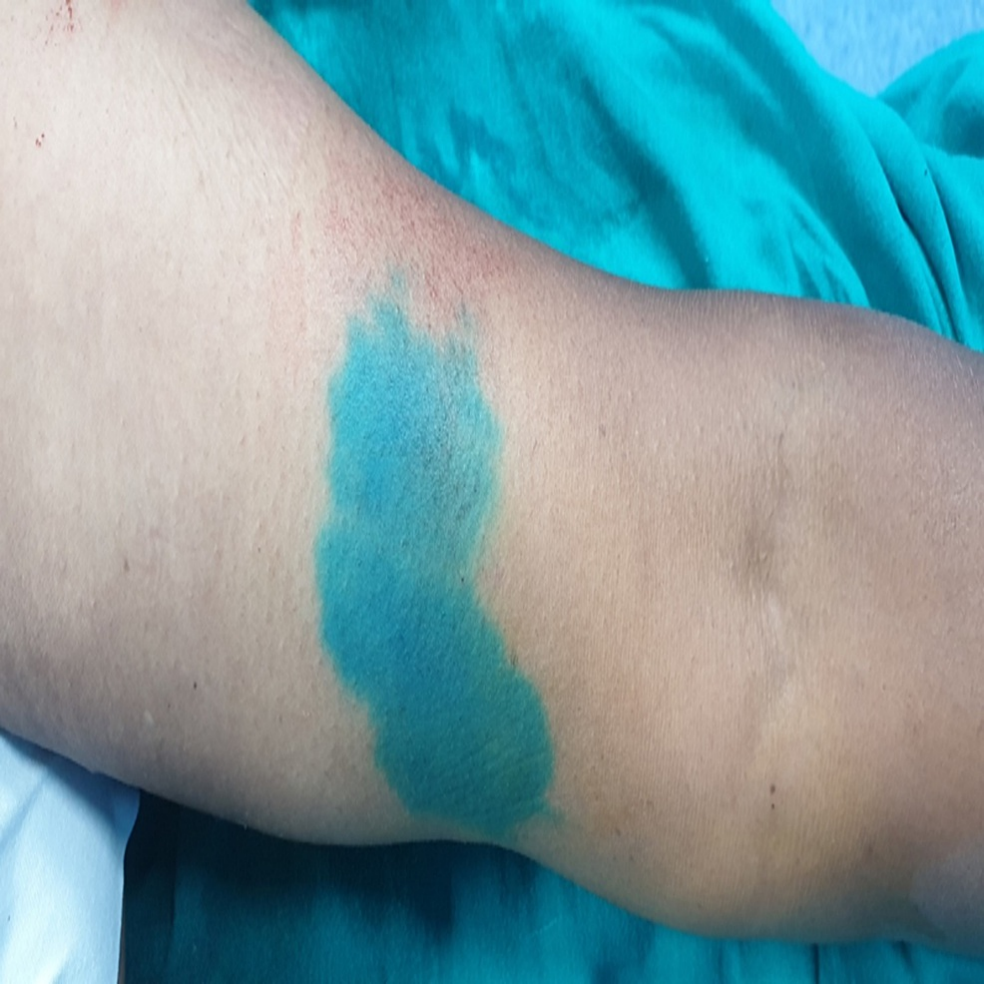
Discoloration leading to temporary tattooing

The complication present was discoloration in about 53% of the patients; there was temporary tattooing of the injection site in a minority of the cases as shown in Figure 4, which resolved itself without any intervention, and there was no permanent tattooing in any cases.

## Conclusions

As determined by this study, the procedure of axillary reverse mapping using isosulfan blue dye is a feasible and reliable adjunct to ALND, which can lead to the preservation of upper limb lymphatics. The study also shows that the template to be considered for axillary dissection in low-volume breast cancer disease can be limited, whereas when compared to high-volume disease, the dissection should be extended to achieve regional control of the pathology, as the axillary lymph node draining the upper limb has a significantly high rate of positivity in cases of high nodal status and locally advanced primary tumor. The procedure also presents as a cheap and reliable way to explore the lymphatic anatomy of the axilla, especially in regard to limb drainage, which can be used in a limited resource setting with minimal complications. Further studies are needed to decide on preserving the ARM node during ALND if noted within the dissection field.
